# Editorial: Advances in modern intelligent surgery: from computer-aided diagnosis to medical robotics

**DOI:** 10.3389/frobt.2025.1620551

**Published:** 2025-05-14

**Authors:** Zhe Min, Rui Song, Changsheng Li, Jax Luo

**Affiliations:** ^1^ School of Control Science and Engineering, Shandong University, Jinan, China; ^2^ Department of Medical Physics and Biomedical Engineering, University College London, London, United Kingdom; ^3^ School of Mechatronical Engineering, Beijing Institute of Technology, Beijing, China; ^4^ Massachusetts General Hospital and Harvard Medical School, Boston, MA, United States

**Keywords:** surgical robotics, medical robotics, surgical navigation, deep learning, computer-assisted diagnosis, medical image segmentation, pre-operative planning, medical image registration

## 1 Introduction

Medical robots enable accurate, safe and minimally invasive treatments of patients in modern operating rooms, through surgical navigation that spans the entire surgical flow, encompassing the pre-operative surgical planning, intraoperative guidance, and postoperative evaluation (Ciuti et al., 2025; Li et al., 2024). Researchers have investigated all relevant aspects towards this goal (Zhang et al., 2024; Min et al., 2021; Min et al., 2023). In addition, deep-learning techniques have recently demonstrated good performances for various tasks in the intelligent surgical/medical robotic systems, including medical image segmentation (Min et al., 2024a), computer-assisted diagnosis (Yan et al., 2025), medical image registration (Du et al., 2024; Zhang et al., 2024), surgical instrument segmentation, surgical step recognition, etc.

In this Research Topic, we aim to present the latest developments and advances in the intelligent modern surgical system, including computer-assisted diagnosis, surgical navigation and medical robotics. As a result of the call for participation, five papers were finally accepted and collected in this Research Topic.

## 2 Overview of the contents of the Research Topic

In the paper “*Technologies evolution in robot-assisted fracture reduction systems: a comprehensive review*” by Kou et al. the authors comprehensively reviewed essential technologies in robot-assisted fracture reduction systems. The key technologies include the preoperative planning methods of bone fracture reduction (that is, manual, semi-automatic and automatic), intraoperative registration and navigation including no preoperative images registration techniques, 2D/3D image-based registration, 3D/3D image-based registration, image fusion-based registration (for example, the non-rigid ICP algorithm), and the two types of fracture reduction robots including robots based on external fixators, robots for distraction and reduction. In addition, the challenges of developing fracture reduction systems and future promising research trends such as developing automatic preoperative planning methods and real-time estimation of intraoperative reduction force, are discussed.

The following two articles focus on the navigation aspects of medical robotic systems. The paper “*X-ray fluoroscopy guided localization and steering of miniature robots using virtual reality enhancement*” by Alabay et al. presents a proof-of-concept study for the real-time detection, tracking, accurate localization and steering control of miniature robots under X-ray fluoroscopy, with experiments conducted within a static two-dimensional phantom testbed, demonstrates a promising future of three-dimensional navigation and control for miniature robots. The virtual reality environment that plays a key role in the successfully enabled capabilities, being the digital twin of the robot and operational workspace, is synchronized with the real physical workspace using data like the robot position information from the image stream. The paper “*A navigated, robot-driven laser craniotomy tool for frameless depth electrode implantation. An in-vivo recovery animal study*” by Winter et al. introduces a frameless robot-driven laser tool for depth electrode implantation, enabled by pre-operative planning and registration procedures, which has been tested in an *in vivo* recovery animal study for the first time. In addition, the postoperative imaging has been performed to verify the accuracy of the entry and target points, and trajectory angulations for the implanted depth electrodes, while histopathological analysis has been conducted examine the tissues changes on the bone, dura and cortex samples.

Other two contributions aim to address the computer-assisted diagnosis and intervention of bladder cancer using artificial intelligence (AI), respectively. The paper “*Enhancing bladder cancer diagnosis through transitional cell carcinoma polyp detection and segmentation: an artificial intelligence powered deep learning solution*” by Borna et al. systematically compares several deep-learning architectures, including Unet++_vgg19, Unet_vgg11 and FPN_resnet, on the accurate segmentation task of bladder tumor particularly transitional cell carcinoma (TCC) polyps in cystoscopy images. The experimental results with satisfactory performances demonstrate the great potentials of deep-learning models being practically integrated into the current diagnostic workflow, even with low-quality cystoscopy images. The paper “*Automated surgical step recognition in transurethral bladder tumor resection using artificial intelligence: transfer learning across surgical modalities*” by Deol et al. presents the automated surgical step recognition (SSR) model, that classifies the three distinct surgical steps including the primary endoscopic evaluation, the resection of bladder tumor and surface coagulation and hemostasis, specifically for the transurethral resection of bladder tumors (TURBT) procedure. The SSR model that leverages the transfer learning technique is pretrained using laparoscopic videos and fine-tuned on TURBT endoscopic videos, enabling good surgical step recognition performance with a very limited dataset of endoscopic videos.

## 3 Conclusion

The articles collected in this Research Topic provide good demonstrations of how medical imaging and intelligent algorithms could benefit the surgical robotic systems and surgical procedures at the end of the day. Despite the achieved significant progress, several challenges still remain in the on-going research development of intelligent surgical robotic systems. For example, to enable accurate navigation and further robot control for robot-assisted soft-tissue surgery, the real-time deformation of soft tissues need to be accurately modelled and the deformable registration between the preoperative and intraoperative spaces is needed (Min et al., 2024b).
